# Address on Filing Teeth

**Published:** 1850-04

**Authors:** J. G. Curtiss


### ADDRESS ON FILING TEETH.

BY DR. CURTISS.

[Read before the Miss. Valley Association of Dental Surgeons, Sept. 1849.]

*Mr. President and Gentlemen of the Association:*

In the consideration of the subject assigned me, from which to address you on the present occasion, namely, the "filing of the teeth," it cannot be expected to embody much that is calculated to amuse, or that might be considered of a novel character. It has fallen to the lot of other gentlemen, (by the assigning of appropriate subjects,) to excite our imagination, call forth our reason, and by their graceful oratory delight our fancy. But in point of practical utility, the subject now before us must be allowed a place in the foremost rank; and although we may not be able to throw around it much embellishment, yet its value as a remedial agent, confirmed by long experience, its almost constant demand, as well as the strong prejudice it has excited, renders it a subject of increasing interest to every member of this association. In almost every instance where persons suppose themselves capable of judging of the qualifications of one of our profession, the first attempt at investigation or inquiry instituted, is to ascertain the dentists views in reference to the use of the file upon the teeth, and as he judges in this one particular, so they judge of his whole professional "modus operandi," and the acceptance or rejection of his services are thus hastily and irrevocably sealed. Does this, gentlemen, seem like strong language, and the implying of a strong popular prejudice? we admit it, but facts justify it. Such prejudice does exist. And some professed dentists, lacking the moral courage to meet this ill-founded prejudice, have ignobly cowered before it, and in opposition to their better judgment, and the true interest of their patrons, have wholly renounced the use of the file upon the teeth, that they might thereby ingratiate themselves into public favor. Such persons are traitors to reason and common honesty, and are richly deserving the scorn of every philanthropist, and all lovers of science.

Nor has the opposite extreme lacked for votaries also. For others have fallen into the erroneous supposition that irritation produced by contact of the teeth is the principal cause of caries, and hence have heroically set themselves to saw assunder, and that indiscriminately all sound teeth, with the *very* wise intention to nip disease a little before its budding. Yet these erudite philosophers have forgotten the scripture maxim that the whole need not a physician.

But while we thus mark out the errors of extremes, we would not wish to be understood as discountenancing the proper use of the file. As a remedy it is invaluable. In using it upon the teeth, however, much discrimination and judgment are necessary. The mouth may be in a condition in which the use of the file would be quite impracticable if not positively injurious. For instance, when from decayed or aching teeth, or the presence of salivary calculus, the gums have assumed a vicious habit, filing would then be liable to increase the inflammation, causing pain, and perhaps even the formation of an abscess. In such cases remedies should be used to restore the mouth to a healthy condition before proceeding with the operation. Again, if a patient of scofulous habit be afflicted with Atrophy of the teeth, (a disease which makes its appearance on the labial surface of the teeth, and is generally sore to the touch,) in such a case filing would be attended with much pain and an increased susceptibility to heat and cold, and perhaps, weeks would elapse before the irritation could be allayed, which would render an operation impracticable. Immediate effects make the strongest impressions upon a patient or upon a community, hence, it is better (in general) to forego an operation than to disappoint their hopes, or, (for any considerable time,) to leave them in suspense. For they are sure to forebode evil. I speak with reference to the dentists' reputation, which should not be altogether lost sight of. Yet by far the greater cause of complaint is not that a tooth is filed little or much, but it is the unskilful manner in which it is performed. The operator is too apt to imagine himself in a hurry and to apply unnecessary force to the file, thereby causing great annoyance, and sometimes serious injury to his patient. Having been under the hands of numerous operators, and some of whom were eminent in the profession, I have the more confidence in the correctness of the assertion, that the filing of the teeth is rarely performed with sufficient carefulness.

I have also had patients who had been in the hands of other operators, and had such a perfect horror of filing, that the bare mention of it would make them shudder. But by using a sharp file, making the passes slow and lightly, and dipping the file often in water, I could use it freely, without any complaint from the patient, and at the close of the operation, have often had their warmly expressed approbation. And I think you will agree with me in the assertion, that the dentist who uses a dull file and then applies force to hasten the operation, "is penny wise and pound foolish." With regard to the variety of files used upon the teeth, we suppose each of you are so familiar, as to render a description more tedious than profitable.

Having thus briefly introduced the subject to the consideration of this convention, of which we think a more lengthened article would not be as interesting as discussion by a committee of the whole, especially as our standard authors have treated the subject so elaborately, and yet so specific. With a desire therefore, to listen to the various opinions, and practice, upon a subject of so much interest, to your further consideration it is respectfully submitted.

Your very obedient
J. G. CURTISS.

